# Double-Blinded Prospective Randomized Clinical Trial Comparing Regular and Moses Modes of Holmium Laser Lithotripsy

**DOI:** 10.1089/end.2019.0695

**Published:** 2020-05-14

**Authors:** Ahmed Ibrahim, Mostafa M. Elhilali, Nader Fahmy, Serge Carrier, Sero Andonian

**Affiliations:** Division of Urology, Department of Surgery, McGill University, Montreal, Canada.

**Keywords:** laser lithotripsy, ureteroscopy, randomized clinical trial, holmium laser, technology assessment, outcomes assessment

## Abstract

***Objective:*** To compare regular and Moses modes of holmium laser lithotripsy during ureteroscopy in terms of fragmentation/pulverization and procedural times in addition to perioperative complications.

***Patients and Methods:*** After obtaining ethics approval, a prospective double-blinded randomized trial was conducted for patients undergoing holmium laser lithotripsy during retrograde ureteroscopy. Patients were randomly assigned to either regular or Moses modes. Patients and surgeons were blinded to the laser mode. Lumenis 120W generator with 200 Moses D/F/L fibers were used. Demographic data, stone parameters, perioperative complications, and success rates were compared. The degree of stone retropulsion was graded on a Likert scale from 0—no retropulsion to 3—maximum retropulsion.

***Results:*** A total of 72 patients were included in the study (36 per arm). Both groups were comparable in terms of age and preoperative stone size (1.4 cm *vs* 1.7 cm, *p* > 0.05). When compared with the regular mode, Moses mode was associated with significantly lower fragmentation/pulverization time (21.1 minutes *vs* 14.2 minutes; *p* = 0.03) and procedural time (50.9 minutes *vs* 41.1 minutes, *p* = 0.03). However, there were no significant differences in terms of lasing time (7.4 minutes *vs* 6.1 minutes, *p* > 0.05) and total energy applied to the stones (11.1 kJ *vs* 10.8 kJ, *p* > 0.05). Moses mode was associated with significantly less retropulsion (mean grade was 1.0 *vs* 0.5, *p* = 0.01). There were no significant differences between both modes in terms of intraoperative complications (11.1% *vs* 8.3%, *p* > 0.05), with one patient requiring endoureterotomy for stricture in the Moses group. Success rate at the end of 3 months was comparable between both groups (83.3% *vs* 88.4%, *p* > 0.05).

***Conclusion:*** Moses technology was associated with significantly lower fragmentation/pulverization and procedural times. The reduced fragmentation/pulverization time seen using Moses technology could be explained by the significantly lower retropulsion of stones during laser lithotripsy.

## Introduction

Prevalence of urolithaisis has been dramatically increasing in North America. Therefore, it is not surprising that ureteroscopic management of symptomatic upper urinary tract stones has drastically increased up to 250% in the United States.^1,2^ This could be attributed to widespread availability of Holmium laser generators that have the ability to fragment all stone types, leading to high stone-free rates while maintaining a safety profile.^3^

The latest high-power 120W Holmium laser generator from Lumenis (LP120H Moses) is equipped with Moses technology, which divides the holmium laser pulse into two adjacent peaks so that the first peak separates the fluid ahead of the stone (Moses effect), and the second peak is easily and precisely delivered toward the target stone, thus less energy is lost and the laser transmission is less dependent on fiber–stone distance. This reshaped pulse together with its compatible Moses fibers has been shown in both *in vitro* and *in vivo* studies to improve stone fragmentation.^4^ *In vitro* studies showed that Moses technology was associated with significantly higher stone ablation volumes and significantly lower stone retropulsion than the conventional regular mode during Holmium laser lithotripsy.^4,5^ In addition, Moses mode was associated with significantly lower procedural time and significantly improved stone fragmentation efficiency than the regular mode.^6^ However, there are no randomized clinical studies evaluating the effectiveness of this new Moses technology in reducing stone retropulsion and improving procedural and/or fragmentation/pulverization times during ureteroscopic Holmium laser lithotripsy. Therefore, the objective of this clinical trial was to compare regular and Moses modes of Holmium laser lithotripsy in terms of stone retropulsion, stone fragmentation/pulverization time, and procedural time during retrograde ureteroscopy in a double-blinded prospective randomized manner.

## Methods

After obtaining ethics approval, a prospective double-blinded randomized clinical trial was conducted for patients undergoing retrograde ureteroscopy and holmium laser lithotripsy between February 2017 and December 2018. Patients' age and gender together with stone parameters obtained on low-dose noncontrast CT scan were collected. Patients were randomly assigned to have holmium laser lithotripsy with either regular or Moses modes while keeping the other laser settings (J and Hz) comparable. The holmium laser generator (Lumenis Pulse™ 120H) was set to an energy level of 0.4 J and rate of 80 Hz for stone pulverization (stone dusting) and 1.0 J and rate of 10 Hz for stone fragmentation. When possible, ureteral stones were fragmented and basketed out while renal stones were pulverized. Patients and surgeons were blinded to the laser mode (regular *vs* Moses). This was accomplished by turning away the laser generator monitor so that only the research assistant could enter the settings. All procedures were performed by four experienced urologists. Lumenis 120W generator (LP120H Moses) with Moses 200 D/F/L fibers were used for all cases.

For each case, the degree of stone retropulsion was graded by the surgeon on a Likert scale from 0—no retropulsion to 3—maximum retropulsion. Procedural time was measured as the time from introduction of the ureteroscope till the final removal of the ureteroscope. Fragmentation/pulverization time was measured from starting lasing till the end of lasing including laser pedal pauses. Fragmentation/pulverization time could be affected by stone retropulsion or migration during laser lithotripsy. The laser generator measured the lasing time from the start of lasing till the end of lasing. Total energy required to fragment the stone was also measured by the laser generator. Laser lithotripsy-related complications such as presence of ureteral mucosal injury and perforation were classified according to the modified Clavien grading system and compared between the two groups.^6^ Success rate was assessed using low-dose CT scans at 3 months. Success rate was defined as no ureteral fragments or presence of nonobstructive renal fragments of ≤4 mm.^7^

### Sample size calculation

The sample size calculation was performed using the G* Power 3.1.9.2 for Windows, which was downloaded from the website (www.gpower.hhu.de) and was accessed on November 10, 2016. A total sample size of 72 ureteroscopy procedures (36 for each group) were required based on employing the independent sample (*t*) test to compare between two groups, with a one-tailed *α* error of 0.05, effect size of 0.6, power (1 − *β* error) of 0.8, and allocation ratio (N2/N1) of 1. This yielded an overall sample size of 72 patients (36 per arm).

### Randomization

A stratified block randomization was used depending on total stone size measured on preoperative CT scan of the abdomen and pelvis. Computer-generated random tables in a 1:1 ratio were used. Patients were randomly assigned to one of two treatment groups (regular *vs* Moses) by stratified–blocked randomization across two strata derived from predetermined stone size grouping (<10 and ≥10 mm).

### Statistical analysis

Data were collected and tabulated using the commercially available SPSS software version 21 (SPSS, Inc., Chicago, IL). Descriptive statistics were presented in terms of percentages and means. Differences between both groups were compared using Fisher's exact test for categorical variables and Student's *t*-test or Mann–Whitney *U*-test to compare normally and abnormally distributed continuous variables, respectively. Two-tailed *p*-values of <0.05 were set for statistical significance.

## Results

**FIG. 1.**
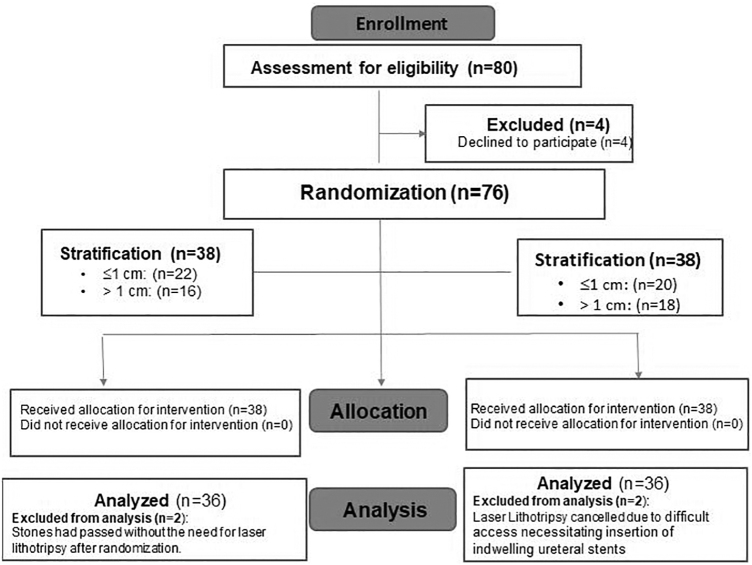
CONSORT (Consolidated Standards of Reporting Trials) flowchart for the study.

A total of 72 patients were included in the final analysis in this double-blinded prospective randomized clinical trial (36 patients in each arm) between February 2017 and December 2018 (Fig. 1). Both modes were compared using 1 J and 10 Hz for fragmentation and 0.4 J and 80 Hz for pulverization settings. When possible, ureteral stones were fragmented and basketed out while renal stones were pulverized. Both groups were comparable in terms of mean age and mean total preoperative stone size (1.4 cm *vs* 1.7 cm, *p* > 0.05) (Table 1). Similarly, both groups were comparable in number of stones, location, opacity, and Hounsfield unit (*p* > 0.05) (Table 1). Both groups were also comparable in preoperative ureteral stenting (41.7% *vs* 27.7%, *p* = 0.06) and degree of hydronephrosis (*p* = 0.2) (Table 1). There was no significant difference in the percentage of cases performed with a ureteral access sheath (38.9% *vs* 30.6%, *p* = 0.08).

**Table 1.** Stone Parameters, Intra- and Postoperative Characteristics

Parameters	Regular mode (*n* = 36)	Moses mode (*n* = 36)	p
Mean age (years)	54.7 ± 13.6	57.4 ± 11.9	0.4
Mean stone size (cm)	1.4 ± 0.97	1.7 ± 1.5	0.2
Mean Hounsfield units	841 ± 348	991 ± 213	0.4
Stone opacity, *n* (%)			
Opaque	33 (91.7)	31 (86.2)	0.9
Lucent	3 (8.3)	5 (13.8)	
Number of stones, *n* (%)			
Single	17 (47.2)	15 (41.7)	0.6
Multiple	19 (52.8)	21 (58.3)	
Stone location, *n* (%)			
Upper calix	2 (5.6)	1 (2.8)	0.5
Lower calix	4 (11.1)	5 (13.8)	
Multiple calices	13 (36.1)	10 (27.8)	
Kidney and ureteral	6 (16.7)	7 (19.4)	
Proximal ureteral	8 (22.2)	9 (25)	
Distal ureteral	3 (8.3)	4 (11.1)	
Stone laterality, *n* (%)			
Right	13 (36.1)	9 (25)	0.2
Left	22 (61.1)	25 (69.4)	
Bilateral	1 (2.8)	2 (5.6)	
Stone composition, *n* (%)			
Calcium oxalate	7 (19.4)	9 (25)	0.2
Magnesium ammonium phosphate	2 (5.6)	3 (8.3)	
Uric acid	3 (8.3)	4 (11.1)	
Cystine stone	0 (0)	1 (2.8)	
Calcium oxalate and calcium phosphate	12 (33.3)	10 (27.8)	
Not available	12 (33.3)	9 (25)	
Degree of hydronephrosis, *n* (%)			
No hydronephrosis	5 (13.9)	4 (12.2)	0.2
Mild hydronephrosis	21 (58.3)	23 (63.9)	
Moderate-to-severe hydronephrosis	10 (27.8)	9 (24.9)	
Preoperative ureteral stenting, *n* (%)			
Yes	15 (41.7)	10 (27.7)	0.06
No	21 (58.3)	26 (72.2)	
Intraoperative access sheath			
Yes, *n* (%)	14 (38.9)	11 (30.6)	0.08
No, *n* (%)	22 (61.1)	25 (69.4)	
Mean lasing time (minutes)	7.4 ± 5.7	6.1 ± 9.8	0.60
Mean fragmentation/pulverization time (minutes)	21.1 ± 15.1	14.2 ± 12.3	0.03^*^
Mean procedure time (minutes)	50.9 ± 27.9	41.1 ± 21.1	0.03^*^
Mean total energy (kJ)	11.1 ± 20.3	10.8 ± 14.1	0.9
Mean retropulsion grade	1 ± 0.68	0.5 ± 0.45	0.01^*^
Intraoperative complication, *n* (%)	4 (11.1)	3 (8.3)	0.1
Success rate, %	83.3	88.4	0.1

^*^ indicates the significant values of *p* < 0.05.

When compared with the regular mode, Moses technology was associated with significantly lower fragmentation/pulverization time (21.1 minutes *vs* 14.2 minutes; *p* = 0.03) and procedural time (50.9 minutes *vs* 41.1 minutes, *p* = 0.03) despite using similar amount of energy (11.5 kJ *vs* 10.8 kJ, *p* > 0.05) (Table 1). When compared with the regular mode, Moses technology was associated with significantly less retropulsion (mean grade 1.0 *vs* 0.5, *p* = 0.01) (Table 1). Success rates at the end of 3 months were comparable between both groups (83.3% *vs* 88.4%, *p* > 0.05) (Table 1).

Intraoperative complications were comparable between both groups (11.1% *vs* 8.3%, *p* > 0.05). However, the sixth randomized patient to the Moses group had a small ureteral perforation during laser lithotripsy of an impacted 1.4 cm ureterovesical junction stone. The patient had undergone previous open radical prostatectomy followed with external beam radiation for recurrent prostate cancer. At the beginning of the operation, a hydrophilic guidewire could not be passed beyond the impacted stone. Holmium laser was used to pulverize the stone using 0.4 J and 80 Hz settings and open up a channel to place the guidewire. At the end of laser lithotripsy, ureteral perforation was suspected. A retrograde ureterogram was performed and showed extravasation of contrast. At this point, ureteral stent was inserted and retrieved after 1 month. During the follow-up, the patient developed a distal ureteral stricture, which was effectively treated with endoureterotomy (Clavien IIIB). Subsequent to this case, there were four cases (two in each group) wherein lower energies of 0.2 J and 80 Hz were used for pulverization of impacted ureteral stones.

## Discussion

Currently, Holmium laser lithotripsy is the cornerstone for management of symptomatic renal and ureteral stones. However, there are still certain challenges that occasionally lead to increased operative time. Consequently, continuous efforts to integrate new technologies into lithotripters to improve the fragmentation efficiency and overcome certain challenges are ongoing.^3,8^ One of these new Holmium laser technologies is the “Moses technology,” which was recently introduced by Lumenis. Moses technology was shown to improve Holmium laser lithotripsy in *in vivo* and *in vitro* studies when compared with the conventional regular mode of Holmium laser lithotripsy.^4,5^ However, there are no randomized clinical trials comparing both modes in terms of fragmentation and procedural times in addition to perioperative complications. Therefore, the aim of this study was to compare regular and Moses modes of holmium laser lithotripsy in a double-blinded prospective randomized manner.

Our results revealed that when compared with the regular mode, Moses technology was associated with significantly lower fragmentation/pulverization time (21.1 minutes *vs* 14.2 minutes, *p* < 0.05) and procedural time (50.9 minutes *vs* 41.1 minutes, *p* < 0.05) despite using comparable amounts of total energy (11.1 kJ *vs* 10.8 kJ, *p* > 0.05). This is most likely related to the significantly less retropulsion seen with Moses technology (mean grade 1.0 *vs* 0.5, *p* < 0.05), leading to less pauses during fragmentation/pulverization to adjust the position of the Holmium laser fiber onto the stone.

Intraoperative complications were comparable between both groups. However, one case from the Moses group had a small ureteral perforation during laser lithotripsy. The thermal effect of the Holmium laser during lithotripsy has been examined by several recent studies. Aldoukhi et al. reported the possibility of renal or ureteral injury when the temperature exceeded the threshold for tissue injury in <20 seconds when 0.5 J and 80 Hz were applied in pigs.^9^ Furthermore, Wollin and coworkers reported that there was a correlation between laser settings and irrigation rates on ureteral temperatures during holmium laser lithotripsy in their *in vitro* model.^10^ However, the impact of Moses technology on heat production during lithotripsy has not been reported in the literature.

Moses technology has been investigated in several recent studies. In their *in vivo* and *in vitro* studies, Elhilali et al.^4^ found that Moses technology was associated with greater stone ablation and reduced stone retropulsion than the short pulse of the regular mode of holmium lithotripsy. Using stone simulator setup, Ibrahim and coworkers^6^ reported a significant reduction of the procedural time with the Moses technology when compared with the regular mode of Holmium laser lithotripsy. Furthermore, Keller and associates^11^ reported that the pronounced effects of the Moses technology could be attributed to the photothermal effect of the Holmium laser on stone fragmentation and suggested further studies to address this specific mechanism. Winship et al.^5^ also reported that Moses technology provides greater ablation of soft stones than long pulse lithotripsy. However, in their *in vitro* study, there were no significant differences between Moses technology and other Holmium laser pulse modifications when using hard stones. All of these studies were limited to the preclinical level. This study is the first study to compare both modes in a double-blinded prospective randomized manner.

This study is not without limitations. First, it only compared between Moses technology and short pulse of Holmium laser for the regular mode. The long pulse of regular mode was not studied. Second, a mixture of stone fragmentation and pulverization was used during this study. When possible, ureteral stones were fragmented and basketed out while renal stones were pulverized. Therefore, the inclusion of both renal and ureteral stones in the study could have confounded the results. Finally, this study was performed at a single tertiary care center. Nevertheless, this is the first randomized trial to address the clinical utility of the Moses technology in a double-blind prospective randomized manner and it showed that Moses technology resulted in significantly lower stone retropulsion resulting in significantly shorter fragmentation/pulverization and procedural times. Future studies need to compare the long pulse of the regular mode with the Moses technology in addition to performing cost analysis between both technologies.

## Conclusions

Moses technology was associated with significantly lower fragmentation/pulverization and procedural times. The reduced fragmentation/pulverization time could be explained by significantly lower retropulsion of stones.

## Abbreviation Used

CT: computed tomography
